# Author Correction: Influenza antibody breadth and effector functions are immune correlates from acquisition of pandemic infection of children

**DOI:** 10.1038/s41467-024-52572-3

**Published:** 2024-09-19

**Authors:** Janice Z. Jia, Carolyn A. Cohen, Haogao Gu, Milla R. McLean, Raghavan Varadarajan, Nisha Bhandari, Malik Peiris, Gabriel M. Leung, Leo L. M. Poon, Tim Tsang, Amy W. Chung, Benjamin J. Cowling, Nancy H. L. Leung, Sophie A. Valkenburg

**Affiliations:** 1 HKU-Pasteur Research Pole, School of Public Health, Li Ka Shing Faculty of Medicine, The University of Hong Kong, Hong Kong, SAR China; 2 Division of Public Health Laboratory Sciences, School of Public Health, Li Ka Shing Faculty of Medicine, The University of Hong Kong, Hong Kong, SAR China; 3 Department of Microbiology and Immunology, Peter Doherty Institute for Infection and Immunity, University of Melbourne, Melbourne, VIC Australia; 4 Molecular Biophysics Unit, Indian Institute of Science, Bangalore, India; 5 Centre for Immunology and Infection (C2i), Hong Kong Science and Technology Park, Hong Kong, SAR China; 6 WHO Collaborating Centre for Infectious Disease Epidemiology and Control, Li Ka Shing Faculty of Medicine, The University of Hong Kong, Hong Kong, SAR China; 7 Laboratory of Data Discovery for Health Limited, Hong Kong Science and Technology Park, Hong Kong, SAR China

**Keywords:** Inactivated vaccines, Influenza virus, Paediatric research, Antibodies

Correction to: *Nature Communications* 10.1038/s41467-024-47590-0, published online 13 April 2024

The original version of this Article contained an error in the final paragraph of page 5, second to last sentence in that it contained incorrect *p*-values for Figure 3 panel d (*p*-value changed from *p* = 0.0427 to *p* = 0.0373) and panel e (*p*-value changed from *p* = 0.0017 to *p* = 0.0034).

The original version of this Article contained an error in the final paragraph of page 5, last sentence in that it contained an incorrect *p*-value for Figure 3 panel f (*p*-value changed from *p* = 0.0226 to *p* = 0.0367) and that panel f was labelled as panel e.

The original version of this Article contained an error in Figure 3 in that a *p*-value and line was missing above the data points in panel f.

The original version of this Article contained an error in the legend of Figure 3 in that the first sentence did not contain a description for panel b and contained a subsequent incorrect labelling of panels c, d, e and f.

The original version of this Article contained an error in the first sentence of the Acknowledgement section in that a funding source was incorrectly missing.

This has all been corrected in both the PDF and HTML versions of the Article.

