# Supplementary material for: Influenza antibody breadth and effector functions are immune correlates from acquisition of pandemic infection of children
Source: Nat Commun. 2024 Apr 13;15:3210. doi: 10.1038/s41467-024-47590-0 (PMC11016072; doi:10.1038/s41467-024-47590-0)
Supplement: Supplementary file 1 — Supplementary Information [file 41467_2024_47590_MOESM1_ESM.pdf]

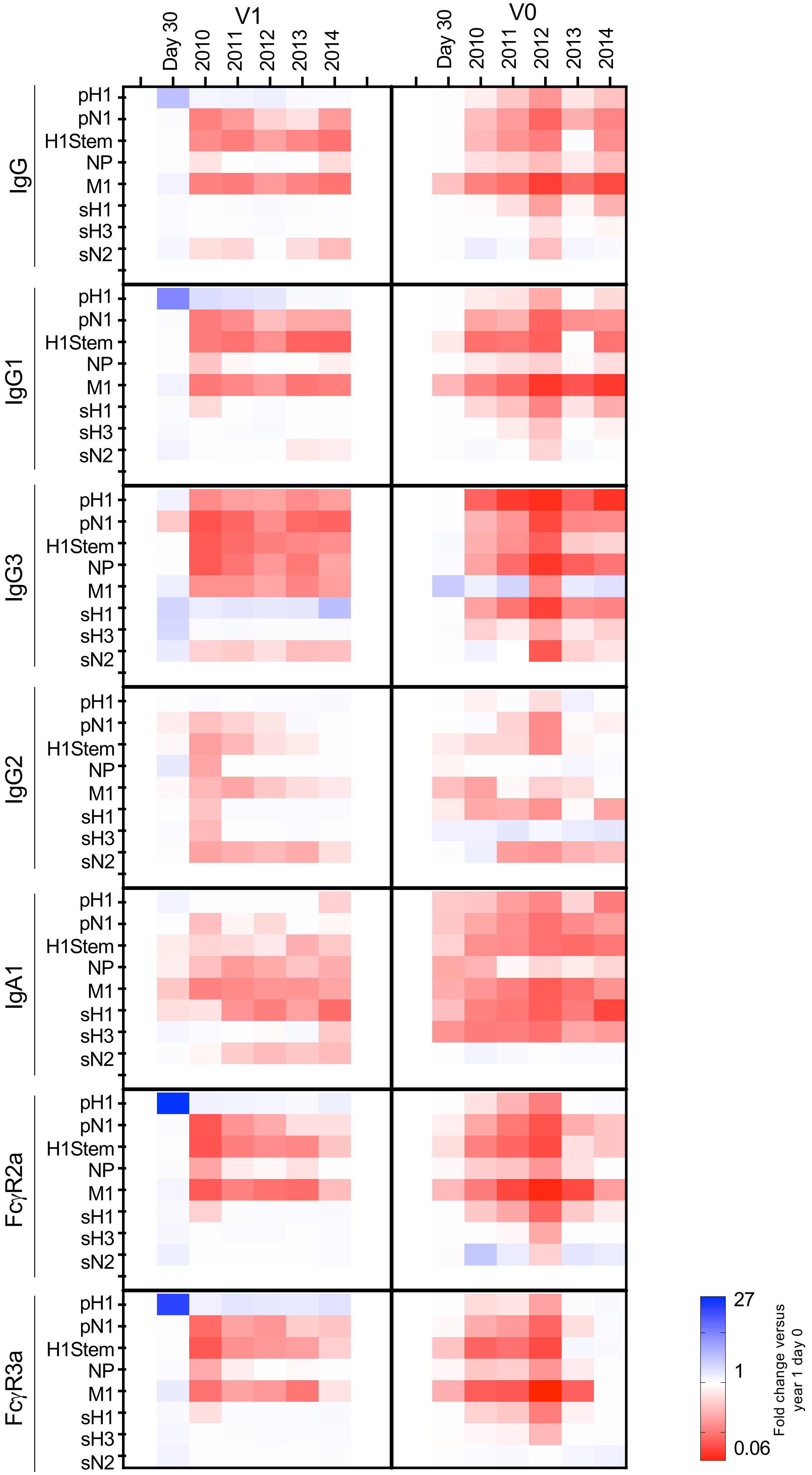

V1
V0
Day 30
2010
2011
2012
2013
2014
IgG
IgG1
IgG3
IgG2
IgA1
FcγR2a
FcγR3a
pH1
pN1
H1Stem
NP
M1
sH1
sH3
sN2
27
1
0.06
Fold change versus year 1 day 0

Supplementary figure 1: Longitudinal fold change of antibodies for seasonal vaccine and pdmH1N1. Children were sampled pre versus post vaccination (V1, n=8, blue) or placebo (V0, n=6, red), and at yearly follow up for 5 years (from Fig. 2, sub-study (ii)). Selected children had no further influenza vaccination or infection during this time. By multiplex bead assay, the MFI fold change versus pre vaccination responses 1 month to 5 years later was assessed. Data represents the group mean fold change. Statistically significant differences shown in Fig. 2.

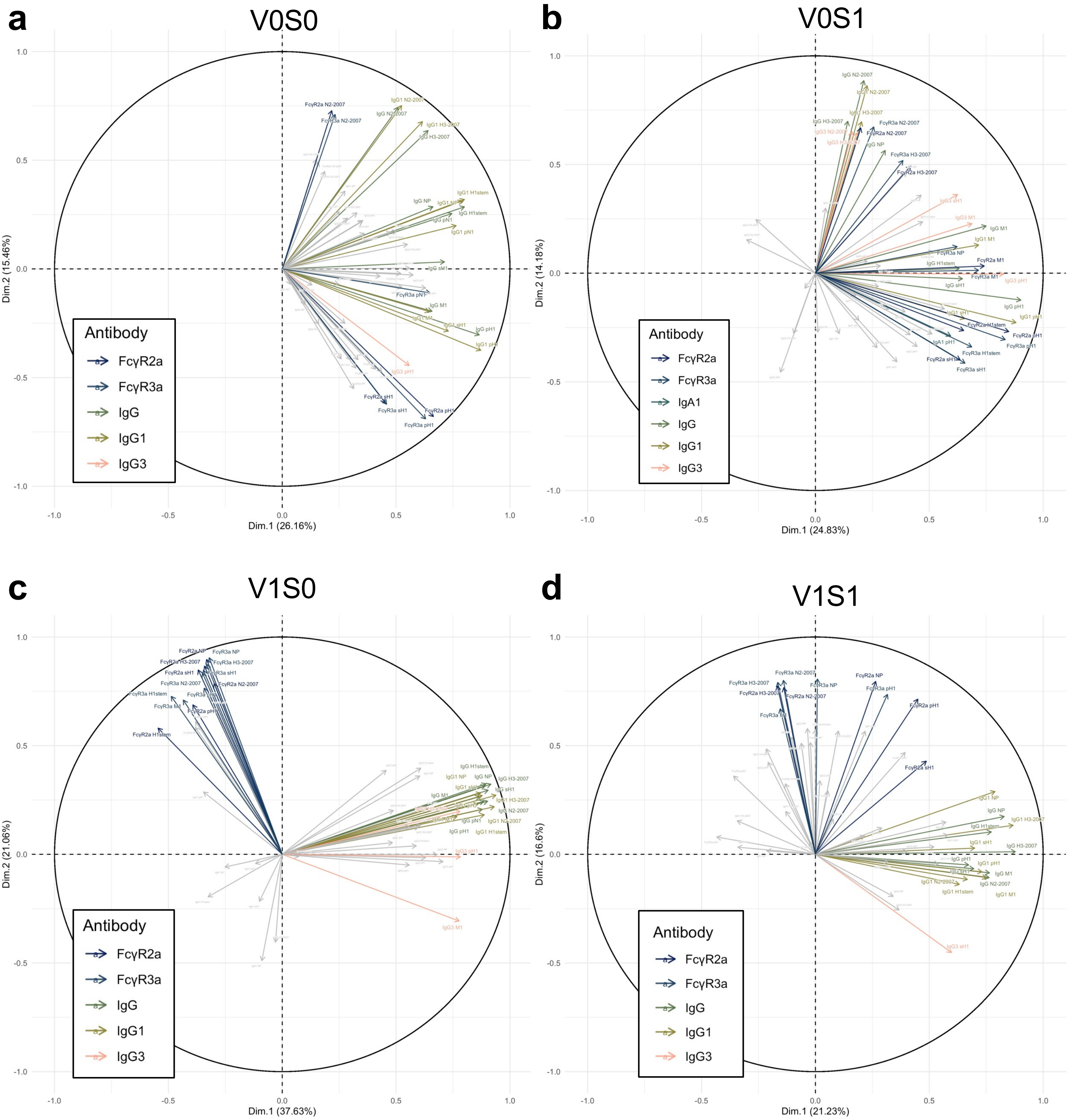

Supplementary figure 2: Baseline drivers for antibody responses in children. From Fig. 4e, day 30 post vaccination pre infection, antibody profiles by principal component analysis. Shaded grey arrows indicate below expected contribution (of the expected value if the contribution were uniform).

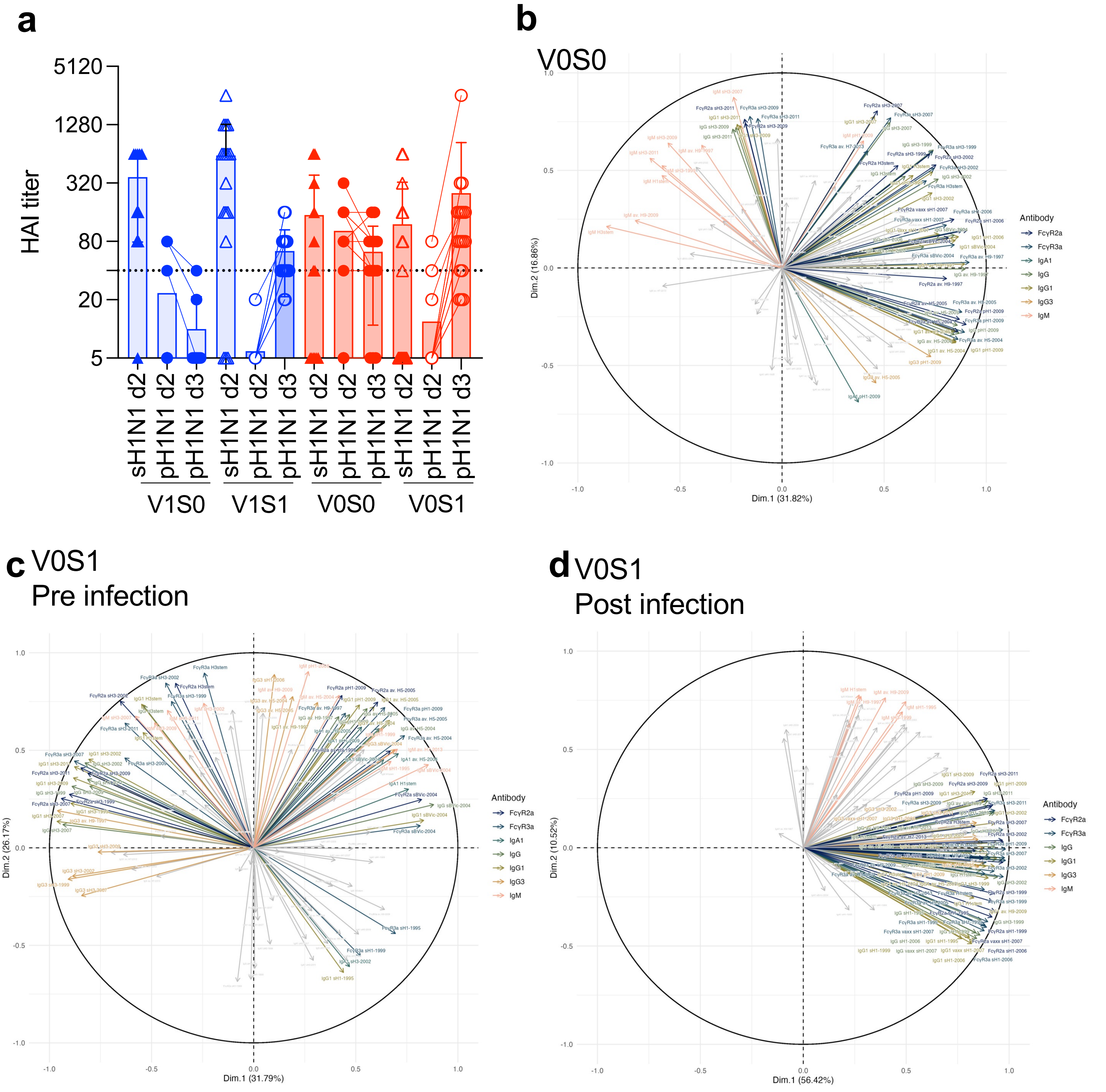

Supplementary figure 3

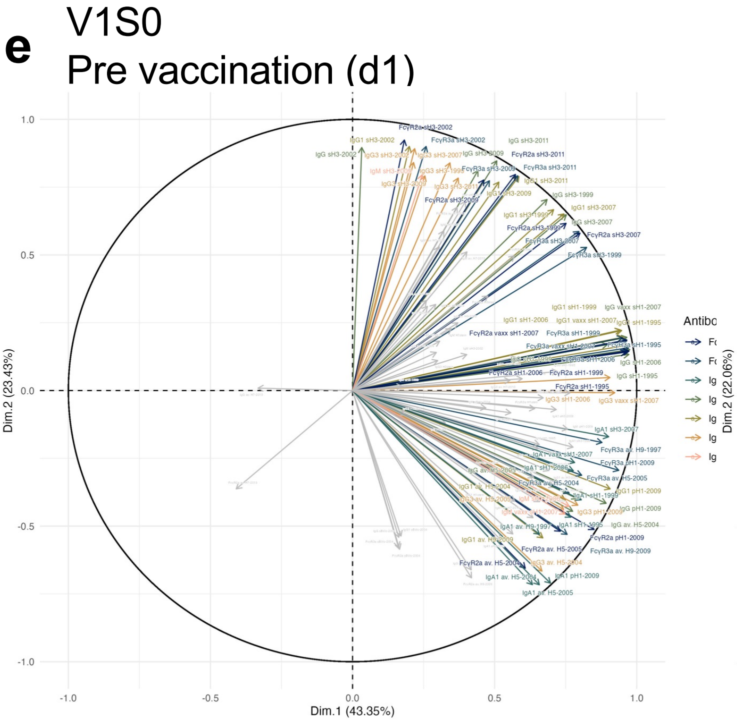

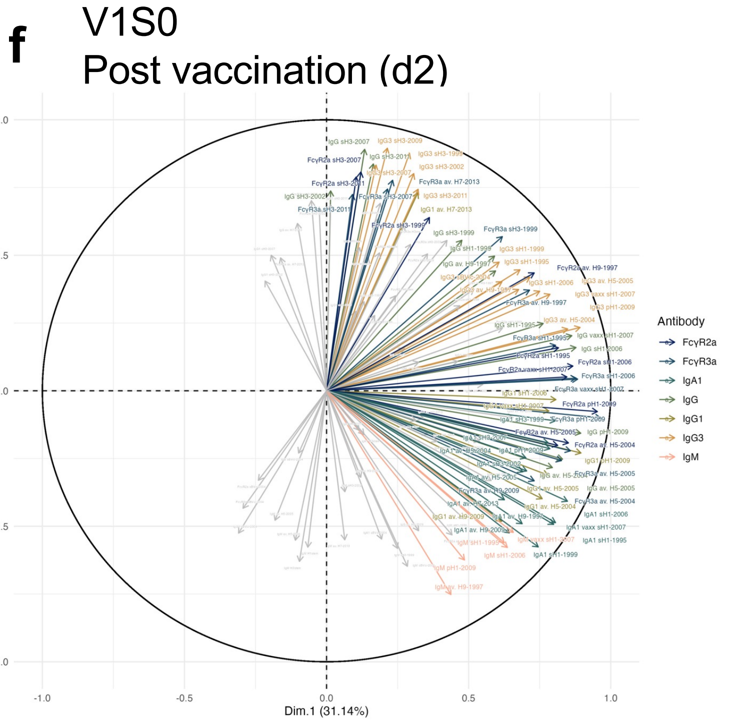

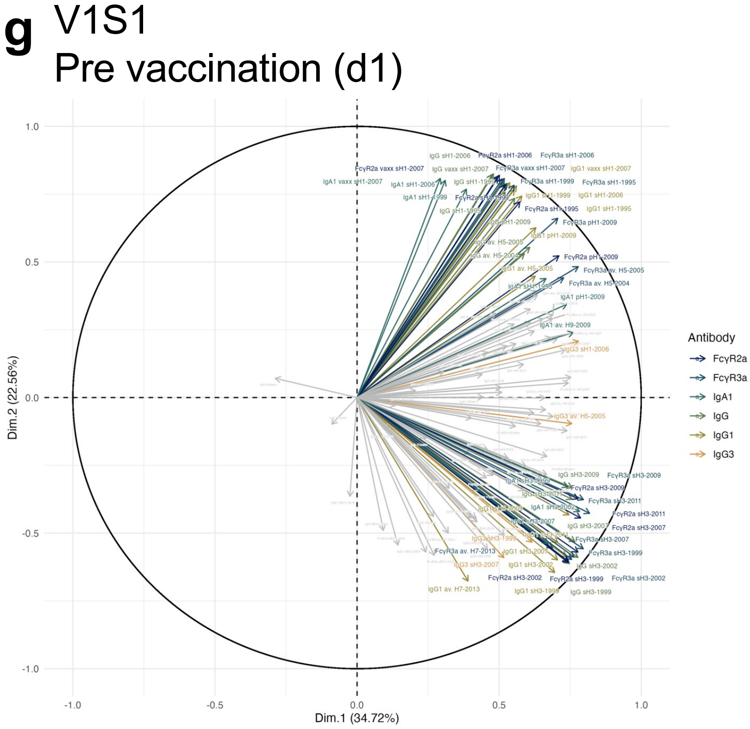

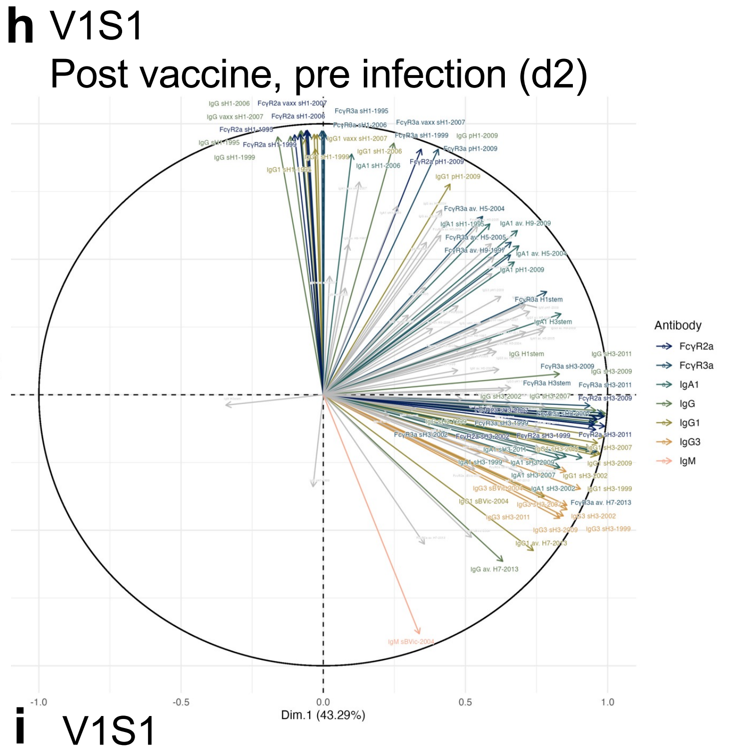

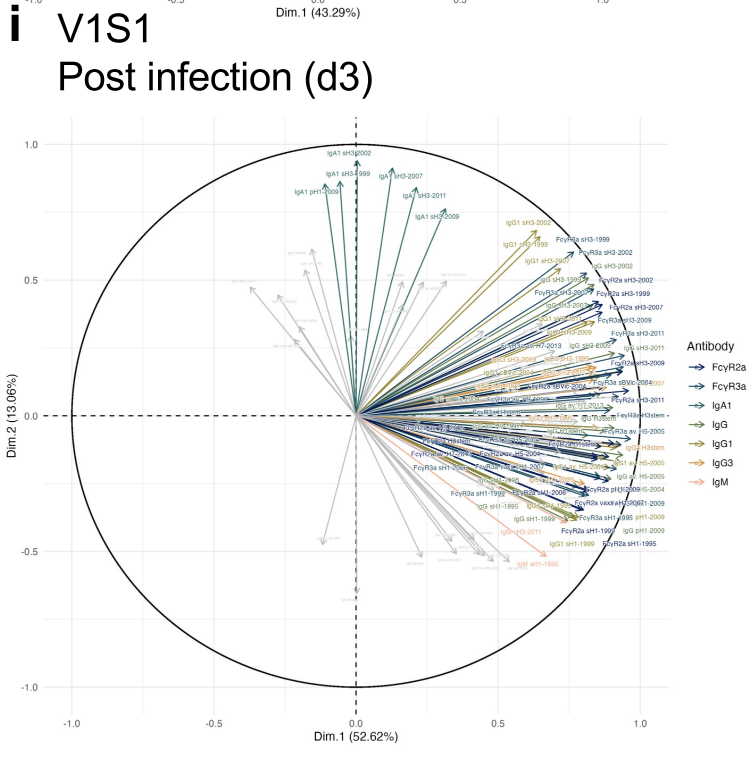

Supplementary figure 3: Seasonal vaccination and pandemic infection for antibody responses in children. Sub-study (iv) From Fig. 5b, antibody profiles by principal component analysis. Shaded grey arrows indicate below expected contribution (of the expected value if the contribution were uniform).

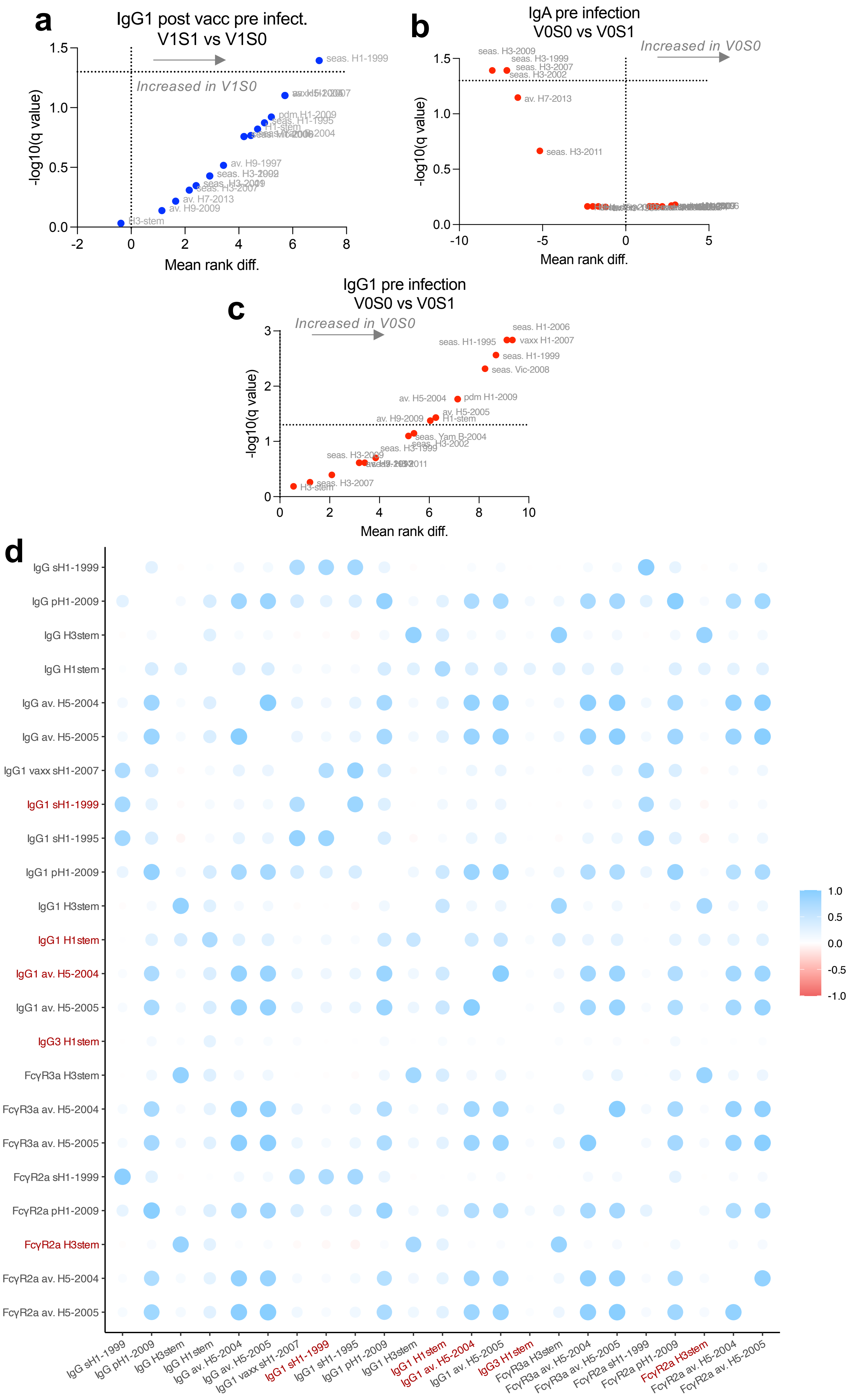
a
IgG1 post vacc pre infect.
V1S1 vs V1S0
Increased in V1S0
-log10(q value)
Mean rank diff.
seas. H1-1999
pdm H1-2009
seas. H1-1995
H1-stem
av. H9-1997
av. H7-2013
av. H9-2009
H3-stem
b
IgA pre infection
V0S0 vs V0S1
Increased in V0S0
-log10(q value)
Mean rank diff.
seas. H3-2009
seas. H3-1999
seas. H3-2007
seas. H3-2002
av. H7-2013
seas. H3-2011
c
IgG1 pre infection
V0S0 vs V0S1
Increased in V0S0
-log10(q value)
Mean rank diff.
seas. H1-2006
seas. H1-1995
vaxx H1-2007
seas. H1-1999
seas. Vic-2008
av. H5-2004
pdm H1-2009
av. H5-2005
av. H9-2009
H1-stem
seas. Yam B-2004
seas. H3-2002
seas. H3-1999
seas. H3-2009
seas. H3-2007
H3-stem
d
IgG sH1-1999
IgG pH1-2009
IgG H3stem
IgG H1stem
IgG av. H5-2004
IgG av. H5-2005
IgG1 vaxx sH1-2007
IgG1 sH1-1999
IgG1 sH1-1995
IgG1 pH1-2009
IgG1 H3stem
IgG1 H1stem
IgG1 av. H5-2004
IgG1 av. H5-2005
IgG3 H1stem
FcγR3a H3stem
FcγR3a av. H5-2004
FcγR3a av. H5-2005
FcγR2a sH1-1999
FcγR2a pH1-2009
FcγR2a H3stem
FcγR2a av. H5-2004
FcγR2a av. H5-2005
1.0
0.5
0.0
-0.5
-1.0

Supplementary figure 4: Significance of HA diversity and correlation of antibody features. From Fig. 5, to determine breadth features the mean difference of responses as a volcano plot analysis for V1S1 vs V1S0 IgG1 (a), V0S0 vs V0S1 IgA (b), V0S0 vs V0S1 IgG1 (c) (FDR rate for multiple comparisons, q-value 1.3 as dotted line for significant results by Mann-Whitney test). From Fig. 5d, (d) matrix of Pearson correlation significance, only >0.8 variables with at least one selected variable were shown. The selected variables were highlighted in red in figures.

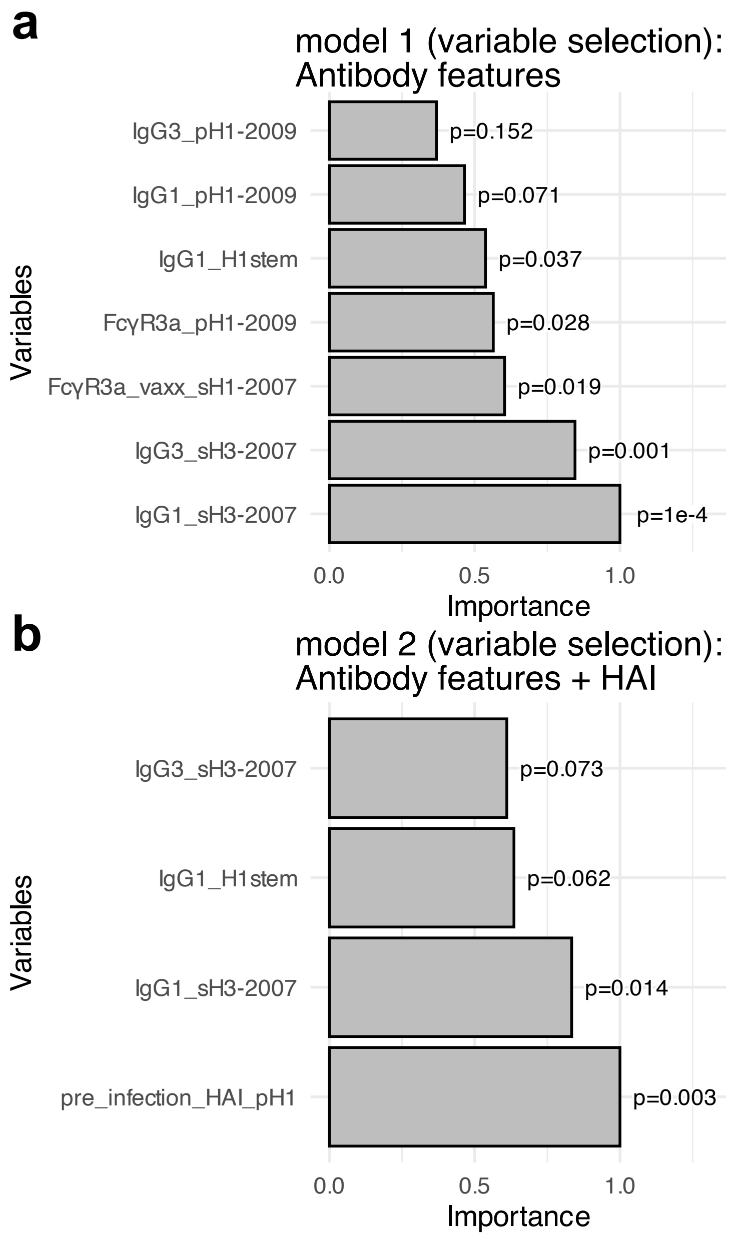

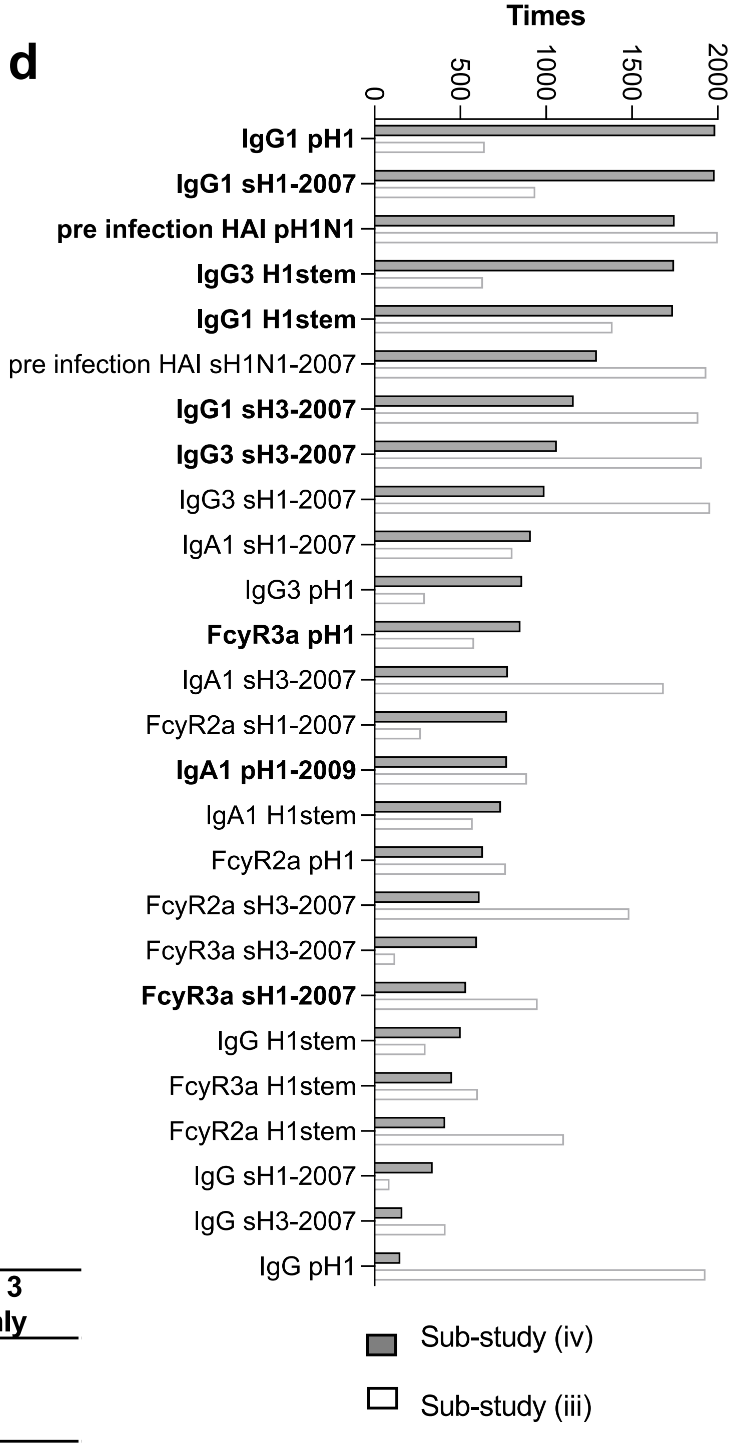

c

Training *(Aim 2 data)*	**Model 1 Antibody**	**Model 2 Antibody+HAI**	**Model 3 HAI only**
Accuracy	0.75	0.76	0.77
Sensitivity	0.71	0.56	0.57
Specificity	0.77	0.94	0.95
Testing *(Aim 3 data)*			
Accuracy	0.36	0.39	0.75
Sensitivity	0	0	0.59
Specificity	0.93	1	1

Supplementary figure 5: Predictive model of antibody features for pandemic infection. (a-c) A logistic regression model with stepwise variable selection by AIC for prediction of infection was built based on day 30 post vaccination/pre infection timepoint, for sub-study (iii) (Fig. 4) data (n=115) (a), and for prediction of sub-study (iv) (Fig. 5) samples infection status (n=36) (b), using the top 10 shared experimental protein/antibody features among 24 variables (a), including 4 proteins (i.e. sH1-2007, pH1, sH3-2007 and H1-stem) in 6 antibody features (i.e. IgG, IgG1, IgG3, FcγR3a, FcγR2a, IgA1), and baseline HAI status (>1:40 HAI+). The top 10 features (in bold) included as training data were selected based on (d) ranking of the features needed to predict infection outcomes from feature selection with Elastic Net and prediction using PLSDA of sub-study (iii) and sub-study (iv) day 30 post vaccination/pre infection samples.

Supplementary table 1: Antibody detectors and Influenza virus protein for antibody effector function assays

Cellular ADCC assay for Figure 1

Detectors	Subtype and virus strain	Protein	Abbreviated
CD107a	H1N1 A/Brisbane/59/2007 *	HA	sH1
	H1N1 A/California/04/2009	HA	pH1
		HA-stem	H1-stem
		NA	pN1
		NP	NP

Multiplex assay for Figure 2, 3 and 4

Detectors	Subtype and virus strain	Protein	Abbreviated
IgG	H1N1 A/Brisbane/59/2007 *	HA	sH1
IgG1	H1N1 A/California/04/2009	HA	pH1
IgG2		HA-stem	H1-stem
IgG3		NA	pN1
IgA1		M1	M1
FcγR2a	H1N1 A/California/07/2009	NP	NP
FcγR3a	H3N2 A/Brisbane/10/2007 *	HA	H3-2007
		NA	N2-2007

Multiplex assay for Figure 5

Detectors	Subtype and virus strain	Protein	Abbreviated
IgG	H1N1 A/Brisbane/59/2007*	HA	sH1-2007
IgG1	H1N1 A/California/04/2009	HA	pH1
IgG3		HA-stem	H1-stem
	H1N1 A/Solomon	HA	sH1-2006
IgM	Islands/3/2006		
	H1N1 A/New	HA	sH1-1999
IgA1	Caledonia/20/1999		
FcγR2a	H1N1 A/Beijing/262/1995	HA	sH1-1995
FcγR3a	H3N2 A/Victoria/361/2011	HA	sH3-2011
	H3N2 A/Brisbane/10/2007*	HA	sH3-2007
	H3N2 A/Perth/16/2009	HA	sH3-2009
	H3N2 A/Fujian/411/2002	HA	sH3-2002
	H3N2 A/Moscow/10/1999	HA	sH3-1999
	H3N2 A/HK/1968	HA-stem	H3-stem
	Vic. B/Malaysia/2506/2004	HA	BVic-2004
	H9N2 A/HK/35820/2009	HA	avH9-2009
	H9N2 A/Chicken/HK/G9/1997	HA	avH9-1997
	H7N9 A/Anhui/2013	HA	avH7-2013
	H5N1 A/Vietnam/1194/2004	HA	avH5-2004
	H5N1 A/Indonesia/5/2005	HA	avH5-2005

*Vaccine content**

Supplementary table 2: Recombinant proteins for antibody assays

Multiplex Assay Reagents	Source	Cat #	Expression system
HA H1N1 A/California/04/2009	Sinobiological	11055-V08H2	HEK 293 cells
HA H1N1 A/Brisbane/59/2007	Sinobiological	11052-V08H	HEK 293 cells
HA H1N1 A/Solomon Islands/3/2006	Sinobiological	11708-V08H	HEK 293 cells
HA H1N1 A/New Caledonia/20/1999	Sinobiological	11683-V08H	HEK 293 cells
HA H1N1 A/Beijing/262/1995	Sinobiological	40133-V08B	Baculovirus-Insect cells
HA H3N2 Victoria/361/2011	Sinobiological	40145-V08B	Baculovirus-Insect cells
HA H3N2 A/Brisbane/10/2007	Sinobiological	11056-V08H	HEK 293 cells
HA H3N2 A/Perth/16/2009	Sinobiological	40043-V08H	HEK 293 cells
HA H3N2 A/Fujian/411/2002	Sinobiological	40488-V08B	Baculovirus-Insect cells
HA H3N2 A/Moscow/10/1999	Sinobiological	40154-V08B	Baculovirus-Insect cells
HA Victoria B/Malaysia/2506/2004	Sinobiological	11716-V08H	HEK 293 cells
HA H9N2 A/Hong Kong/35820/2009	Sinobiological	40174-V08B	Baculovirus-Insect cells
HA H9N2 A/Chicken/ Hong Kong /G9/1997	Sinobiological	40036-V08H	HEK 293 cells
HA H7N9 A/Anhui/1/2013	Sinobiological	40103-V08B	Baculovirus-Insect cells
HA H5N1 A/Vietnam/1194/2004	Sinobiological	11062-V08H1	HEK 293 cells
HA H5N1 A/Indonesia/5/2005	Sinobiological	11060-V08H2	HEK 293 cells
H3-stem A/HK/1968	Ragahavan Varadarjan	Custom	E. coli
H1-stem A/California/04/2009	Ragahavan Varadarjan	Custom	E. coli
NA H1N1 A/California/04/2009	BEI Resources	NR-19234	Baculovirus-Insect cells
NA H3N2 A/Brisbane/10/2007	BEI Resources	NR-43784	Baculovirus-Insect cells
NP H1N1 A/California/07/2009	Sinobiological	40205-V08B	Baculovirus-Insect cells
M1 H1N1 A/California/04/2009	My BioSource	MBS434235	E. coli
anti-IgG-Fab Ab	Merck	MAB1304	Mouse
Tetanus protein	Sigma Aldrich	T3194-25UG	Clostridium tetani
