# Supplementary material for: Influenza antibody breadth and effector functions are immune correlates from acquisition of pandemic infection of children
Source: Nat Commun. 2024 Apr 13;15:3210. doi: 10.1038/s41467-024-47590-0 (PMC11016072; doi:10.1038/s41467-024-47590-0)
Supplement: Supplementary file 3 — Reporting Summary [file 41467_2024_47590_MOESM3_ESM.pdf]

Corresponding author(s): Sophie Valkenburg

Last updated by author(s): Mar 13, 2024

Reporting Summary

Nature Portfolio wishes to improve the reproducibility of the work that we publish. This form provides structure for consistency and transparency in reporting. For further information on Nature Portfolio policies, see our Editorial Policies and the Editorial Policy Checklist.

Statistics

For all statistical analyses, confirm that the following items are present in the figure legend, table legend, main text, or Methods section.

n/a	Confirmed	
☐	☒	The exact sample size (*n*) for each experimental group/condition, given as a discrete number and unit of measurement
☐	☒	A statement on whether measurements were taken from distinct samples or whether the same sample was measured repeatedly
☐	☒	The statistical test(s) used AND whether they are one- or two-sided<br>*Only common tests should be described solely by name; describe more complex techniques in the Methods section.*
☐	☒	A description of all covariates tested
☐	☒	A description of any assumptions or corrections, such as tests of normality and adjustment for multiple comparisons
☐	☒	A full description of the statistical parameters including central tendency (e.g. means) or other basic estimates (e.g. regression coefficient) AND variation (e.g. standard deviation) or associated estimates of uncertainty (e.g. confidence intervals)
☐	☒	For null hypothesis testing, the test statistic (e.g. *F*, *t*, *r*) with confidence intervals, effect sizes, degrees of freedom and *P* value noted<br>*Give P values as exact values whenever suitable.*
☒	☐	For Bayesian analysis, information on the choice of priors and Markov chain Monte Carlo settings
☐	☒	For hierarchical and complex designs, identification of the appropriate level for tests and full reporting of outcomes
☐	☒	Estimates of effect sizes (e.g. Cohen's *d*, Pearson's *r*), indicating how they were calculated

Our web collection on statistics for biologists contains articles on many of the points above.

Software and code

Policy information about availability of computer code

Data collection	Multiplex bead assays were acquired using the MAGPIX system (Luminex, array reader v2.6.1, microplate platform v2.1.15, Bio-Plex manager software v6.2.0.175 from BioRad) following manufacturer's instruction to detect the microspheres and binding of PE fluorescence to calculate a median fluorescence intensity (MFI).<br>NK92 cells were acquired by Flow Cytometry (Attune NxT, Invitrogen)
Data analysis	FACS data was analysed by FlowJo software (v10).<br>Statistical analysis was performed using GraphPad Prism v9 and Excel 16.7 software. Analysis scripts have been deposited at Zenodo under accession code https://zenodo.org/records/10583684.

For manuscripts utilizing custom algorithms or software that are central to the research but not yet described in published literature, software must be made available to editors and reviewers. We strongly encourage code deposition in a community repository (e.g. GitHub). See the Nature Portfolio guidelines for submitting code & software for further information.

Data

Policy information about availability of data

All manuscripts must include a data availability statement. This statement should provide the following information, where applicable:

- Accession codes, unique identifiers, or web links for publicly available datasets
- A description of any restrictions on data availability
- For clinical datasets or third party data, please ensure that the statement adheres to our policy

Deidentified raw data that support this study are included.

Research involving human participants, their data, or biological material

Policy information about studies with human participants or human data. See also policy information about sex, gender (identity/presentation), and sexual orientation and race, ethnicity and racism.

Reporting on sex and gender	Sex and gender were not included in our analysis but recorded at recruitment. Table 1 includes gender distribution of our samples.
Reporting on race, ethnicity, or other socially relevant groupings	Race, ethnicity and other social groupings were not included in our analysis.
Population characteristics	The pandemic H1N1 infection status of subjects was determined by seroconversion during year 1 of the study, and reported as S1 or S0 for group stratification. The seasonal vaccination status was assigned at enrollment as vaccinated (V1) or placebo (V0). Participants were aged 6 to 15 years of age (See table 1)
Recruitment	In this exploratory study, we use serum from a randomised placebo-controlled trial of seasonal trivalent influenza vaccination in children (NCT00792051) conducted at the onset of the 2009 H1N1 pandemic (pH1N1) and monitored for infection. Serologically confirmed pdmH1N1 infection within year 1 of this study based is based on HAI status (≥4-fold GMT rise for seroconverted (S1) for pH1, or not sero converted (S0)) after seasonal TIV vaccination or placebo and post season sampling. Age matched controls for uninfected groups (S0) were selected based on the parameters of the infected groups to ensure similar age and gender balance (Table 1). Results may impacted by antibody waning failing to detect infection between sampling timepoints.
Ethics oversight	Proxy written informed consent was obtained for all participants from their parents or legal guardians, with additional written assent from those ≥8 years of age. The study protocol was approved by the Institutional Review Board of Hong Kong University (UW 08-008), and is a registered clinical trial NCT00792051.

Note that full information on the approval of the study protocol must also be provided in the manuscript.

Field-specific reporting

Please select the one below that is the best fit for your research. If you are not sure, read the appropriate sections before making your selection.

☒ Life sciences ☐ Behavioural & social sciences ☐ Ecological, evolutionary & environmental sciences

For a reference copy of the document with all sections, see nature.com/documents/nr-reporting-summary-flat.pdf

Life sciences study design

All studies must disclose on these points even when the disclosure is negative.

Sample size	No sample size calculation was included but based on maximal available samples with serum volume available from a study archive that met our aim selection criteria, e.g. sub-study (ii), no further IAV infection or vaccination during the study period and for Aim 3 with paired samples pre and post vaccination and post infection. For sub-study (i) and (iii) a sample size of 30 was used based on previous maximal experimental feasibility and statistical significance.
Data exclusions	Data from one protein HA B/Vic-2008 was excluded due to low coupling efficiency
Replication	Experiments were repeated, and in multiplex data sets run in duplicate, as indicated in figure legends.
Randomization	Vaccination status was randomized at enrollment.
Blinding	Samples were not blinded due to known infection outcomes and vaccination status with limited sample size.

Reporting for specific materials, systems and methods

We require information from authors about some types of materials, experimental systems and methods used in many studies. Here, indicate whether each material, system or method listed is relevant to your study. If you are not sure if a list item applies to your research, read the appropriate section before selecting a response.

Materials & experimental systems

n/a	Involved in the study
☐	☒ Antibodies
☐	☒ Eukaryotic cell lines
☒	☐ Palaeontology and archaeology
☒	☐ Animals and other organisms
☐	☒ Clinical data
☒	☐ Dual use research of concern
☒	☐ Plants

Methods

n/a	Involved in the study
☒	☐ ChIP-seq
☐	☒ Flow cytometry
☐	☐ MRI-based neuroimaging

Antibodies

Antibodies used	Purified CD16 antibody (1:100, clone: 3G8), anti-human CD107a-APC (1:50, clone: H4A3) and CD56-PE (1:200, clone: 5.1H11) (all Biolegend)
Validation	Product specification sheets for each antibody describe from the company the expected cell proportion based on cell staining. All antibodies were titrated by us prior to use and single colour florescence controls are acquired for each antibody at the time of data acquisition to ensure antibodies in use are working well.

Eukaryotic cell lines

Policy information about cell lines and Sex and Gender in Research

Cell line source(s)	NK-92-FcgR3a-bearing cell line, provided by Fox Chase Cancer Center
Authentication	Morphology check by microscope, only cells at early passage have been used, CD56 and CD16 positivity and used in a functional assay for CD107a degranulation.
Mycoplasma contamination	In-house mycoplasma testing was performed and confirmed to be negative.
Commonly misidentified lines (See ICLAC register)	None

Clinical data

Policy information about clinical studies

All manuscripts should comply with the ICMJE guidelines for publication of clinical research and a completed CONSORT checklist must be included with all submissions.

Clinical trial registration	NCT00792051.
Study protocol	DOI: 10.1093/cid/cis518, Cowling et al., CID 2012
Data collection	November 2008 to December 2014
Outcomes	Serologically confirmed pdmH1N1 infection within year 1 of this study based is based on HAI status (≥4-fold GMT rise for seroconverted (S1) for pH1, or not sero converted (S0)) after seasonal TIV vaccination or placebo and post season sampling.

Plants

Seed stocks | *Report on the source of all seed stocks or other plant material used. If applicable, state the seed stock centre and catalogue number. If plant specimens were collected from the field, describe the collection location, date and sampling procedures.*

Novel plant genotypes | *Describe the methods by which all novel plant genotypes were produced. This includes those generated by transgenic approaches, gene editing, chemical/radiation-based mutagenesis and hybridization. For transgenic lines, describe the transformation method, the number of independent lines analyzed and the generation upon which experiments were performed. For gene-edited lines, describe the editor used, the endogenous sequence targeted for editing, the targeting guide RNA sequence (if applicable) and how the editor was applied.*

Authentication | *Describe any authentication procedures for each seed stock used or novel genotype generated. Describe any experiments used to assess the effect of a mutation and, where applicable, how potential secondary effects (e.g. second site T-DNA insertions, mosiacism, off-target gene editing) were examined.*

Flow Cytometry

Plots

Confirm that:

☒ The axis labels state the marker and fluorochrome used (e.g. CD4-FITC).

☒ The axis scales are clearly visible. Include numbers along axes only for bottom left plot of group (a 'group' is an analysis of identical markers).

☒ All plots are contour plots with outliers or pseudocolor plots.

☒ A numerical value for number of cells or percentage (with statistics) is provided.

Methodology

Sample preparation	NK92 cells were passaged in rhIL-2 media, below passage 20.
Instrument	NK92 cells acquired by Flow Cytometry (Attune NxT, Invitrogen)
Software	Analysed by FlowJo software (v10).
Cell population abundance	NK92 cell line
Gating strategy	FSC/SSC -> CD56+ -> CD107a+

☐ Tick this box to confirm that a figure exemplifying the gating strategy is provided in the Supplementary Information.

Magnetic resonance imaging

Experimental design

Design type	*Indicate task or resting state; event-related or block design.*
Design specifications	*Specify the number of blocks, trials or experimental units per session and/or subject, and specify the length of each trial or block (if trials are blocked) and interval between trials.*
Behavioral performance measures	*State number and/or type of variables recorded (e.g. correct button press, response time) and what statistics were used to establish that the subjects were performing the task as expected (e.g. mean, range, and/or standard deviation across subjects).*

Acquisition

Imaging type(s)	*Specify: functional, structural, diffusion, perfusion.*
Field strength	*Specify in Tesla*
Sequence & imaging parameters	*Specify the pulse sequence type (gradient echo, spin echo, etc.), imaging type (EPI, spiral, etc.), field of view, matrix size, slice thickness, orientation and TE/TR/flip angle.*
Area of acquisition	*State whether a whole brain scan was used OR define the area of acquisition, describing how the region was determined.*

Diffusion MRI ☐ Used ☐ Not used

Preprocessing

Preprocessing software	*Provide detail on software version and revision number and on specific parameters (model/functions, brain extraction, segmentation, smoothing kernel size, etc.).*
Normalization	*If data were normalized/standardized, describe the approach(es): specify linear or non-linear and define image types used for transformation OR indicate that data were not normalized and explain rationale for lack of normalization.*
Normalization template	*Describe the template used for normalization/transformation, specifying subject space or group standardized space (e.g. original Talairach, MNI305, ICBM152) OR indicate that the data were not normalized.*
Noise and artifact removal	*Describe your procedure(s) for artifact and structured noise removal, specifying motion parameters, tissue signals and physiological signals (heart rate, respiration).*
Volume censoring	*Define your software and/or method and criteria for volume censoring, and state the extent of such censoring.*

Statistical modeling & inference

Model type and settings	*Specify type (mass univariate, multivariate, RSA, predictive, etc.) and describe essential details of the model at the first and second levels (e.g. fixed, random or mixed effects; drift or auto-correlation).*
Effect(s) tested	*Define precise effect in terms of the task or stimulus conditions instead of psychological concepts and indicate whether ANOVA or factorial designs were used.*

Specify type of analysis: ☐ Whole brain ☐ ROI-based ☐ Both

Statistic type for inference<br>(See Eklund et al. 2016)	*Specify voxel-wise or cluster-wise and report all relevant parameters for cluster-wise methods.*
Correction	*Describe the type of correction and how it is obtained for multiple comparisons (e.g. FWE, FDR, permutation or Monte Carlo).*

Models & analysis

n/a	Involved in the study
☐	☐ Functional and/or effective connectivity
☐	☐ Graph analysis
☐	☐ Multivariate modeling or predictive analysis

Functional and/or effective connectivity	*Report the measures of dependence used and the model details (e.g. Pearson correlation, partial correlation, mutual information).*
Graph analysis	*Report the dependent variable and connectivity measure, specifying weighted graph or binarized graph, subject- or group-level, and the global and/or node summaries used (e.g. clustering coefficient, efficiency, etc.).*
Multivariate modeling and predictive analysis	*Specify independent variables, features extraction and dimension reduction, model, training and evaluation metrics.*
